# Self-controlling photonic-on-chip networks with deep reinforcement learning

**DOI:** 10.1038/s41598-021-02583-7

**Published:** 2021-11-30

**Authors:** Nguyen Do, Dung Truong, Duy Nguyen, Minh Hoai, Cuong Pham

**Affiliations:** 1 Posts and Telecommunications Institute of Technology, Hanoi, Vietnam; 2 Stony Brook University, Stony Brook, NY USA; 3 VinAI Research, Hanoi, Vietnam

**Keywords:** Engineering, Mathematics and computing, Optics and photonics

## Abstract

We present a novel photonic chip design for high bandwidth four-degree optical switches that support high-dimensional switching mechanisms with low insertion loss and low crosstalk in a low power consumption level and a short switching time. Such four-degree photonic chips can be used to build an integrated full-grid Photonic-on-Chip Network (PCN). With four distinct input/output directions, the proposed photonic chips are superior compared to the current bidirectional photonic switches, where a conventionally sizable PCN can only be constructed as a linear chain of bidirectional chips. Our four-directional photonic chips are more flexible and scalable for the design of modern optical switches, enabling the construction of multi-dimensional photonic chip networks that are widely applied for intra-chip communication networks and photonic data centers. More noticeably, our photonic networks can be self-controlling with our proposed Multi-Sample Discovery model, a deep reinforcement learning model based on Proximal Policy Optimization. On a PCN, we can optimize many criteria such as transmission loss, power consumption, and routing time, while preserving performance and scaling up the network with dynamic changes. Experiments on simulated data demonstrate the effectiveness and scalability of the proposed architectural design and optimization algorithm. Perceivable insights make the constructed architecture become the self-controlling photonic-on-chip networks.

## Introduction

The streaming of immersive multimedia content, the migration of traditional software applications to the cloud computing platform, the widespread deployment of data mining programs and big data applications, and the broadband access demands have led to the explosive growth of bandwidth consumption^1–3^. The advent of the data-intensive spectrum has created a playground for large-scale photonic switches, which play a pivotal role for the next-generation telecommunication networks. More noticeably, the large-scale photonic switches also create a premise for developing advanced data center networks and the state-of-the-art photonic neural information processing systems. Recently, silicon photonics has emerged as a powerful platform for realizing high-density photonic integrated circuits because silicon photonics can enable the monolithic integration of complex circuits at a reasonable cost and high yield by utilizing the advanced features of complementary metal-oxide-semiconductor manufacturing technology^4–6^.

Some various sizable configurations have been introduced for large-scale silicon photonic switches^7–12^ enabling advancements of broad bandwidth, high transmittance, fast response time, and low power consumption^13,14^. Recently, metasurface and phase change materials have been introduced as promising platforms for the next-generation active and low-loss optics due to their unprecedented ability to control incident electromagnetic fields in the subwavelength regime^15,16^ and agile reconfigurable photonic functionalities owing to adjustable properties to fully manipulate the key features of photons, the information carrier in photonic platforms^17–22^. However, such technologies have not been completely developed for the adequate integration with CMOS technology at the silicon-on-insulator (SOI) wafer-scale level. Hence, currently, most fully programmable and scalable switching fabrics in large-scale silicon photonic switches are constructed primarily from multistage structures by manipulating the phase-shifting control technique, for instance, Mach-Zehnder Interferometers (MZIs)^23–25^, multi-mode interference (MMI) couplers^26,27^, and microring resonators (MRR)^28^. *N*$\times$*N* optical switch fabrics are built up by interconnecting multiple stages of elementary switch cells in an available switching topology via passive waveguide elements or Benes types^29–34^. However, current silicon photonic switches due to having the ability to support a large number of input/output ports (up to $128\times 128$) are being still limited in the bidirectional switching ability without supporting higher dimensions. Furthermore, some approaches have been developed by using multistage structures for wavelength division multiplexing (WDM)^35^, mode division multiplexing (MDM)^36^, or WDM-MDM hybridization systems^37,38^. Although WDM or MDM channels deployed by a single laser can carry a very high bit rate up to 400 Gbps by leveraging external modulation methods^39,40^, currently, they do not timely respond to the extremely high-speed access requirements since data center networks have been exceeded Petabyte capacity^41^. Therefore, today’s information connectivity needs no longer stop at wavelength division multiplexing or modal division multiplexing levels, but the individual channel connectivity will approach the fiber level carrying a large number of guided-mode and wavelength division multiplexing channels. Current technology enables high-performance interconnection between a single-mode fiber with a single-mode silicon waveguide via a diffractive grating coupler^42^ or an edge coupler^43^. Therefore, today’s information connectivity needs no longer stop at wavelength division multiplexing or mode division multiplexing levels, but the individual channel connectivity will approach the fiber level carrying a large number of guided-mode and wavelength division multiplexing channels. In addition, the development of high-density waveguide-level silicon photonic switches with omnidirectional connection ability is an extremely critical problem for the photonic community/society. Multi-degree switching functionality can be realized in a fiber coupler switch by a cumbersome mechanical-optical regime or liquid crystal fiber switch. However, there is a big challenge for inventing the silicon photonic switches that allow multi-degree switching functionalities because the multi-degree switching function needs some complex components such as waveguide crossing elements, multiport switching mechanisms, and specific cross-connect elements for directing the arbitrary optical channels. They have not been exploited yet while the next-generation photonic switches are being looked forward to supporting higher dimensional nodes that are feasible and scalable for the design and deployment of future large-scale photonic on-chip interconnects such as photonic intrachip communication networks and all photonic data center network. Over time, to respond to large topologies like high-speed computational systems and broadband data centers, the photonic networks must be constructed from several fundamental switching units to scale up to the full-mesh logic connectivity. Thus, the requirement of multi-degree connection nodes becomes an indispensably critical issue.

To overcome the current limits, a large-scale PCN in the state-of-the-art needs two critical requirements. The first is a design that can be scalable, reconfigurable, and modulable. In which each switching module is constructed by connecting fundamental photonic waveguide elements enabling the multidimensional switching operation at the single-mode waveguide level. The second is to find out an optimization rounting algorithm for the PCN to work faster and more efficiently.

This work proposes Photonic-on-chip Networks (PCNs) based on the interconnection of the unit cells as compact and high bandwidth silicon photonic switches in a full-grid topology. The significant facilities of the proposed PCN architecture are to support the four-degree switching ability in each photonic switching unit and to allow the simultaneous routing functionality from some arbitrary inputs to outputs with many access ports. To address the first requirement, our novel switch architecture has four distinct input/output directions, which are fundamentally different from the previous designs with only two input/output directions. Having more than two input/output directions is a crucial advantage of our investigation because we are no longer bounded by the linear-chain topology where an optical switch can only be built by lining up photonic chips in a row. A further novelty of our work is the design of optical cross-connect elements for delivering the optical signal from an arbitrary input port to a random output port without interfering and congesting with other input/output channels. These cross-connect elements are essential to attain the non-blocking multidimensional switching function, thus making the proposed silicon switches more outperformed than that of existing bidirectional photonic switches^29–34^. To the best of our knowledge, optical cross-connect components in silicon photonics have not been realized before. By mean of the integration of waveguide cross-connect devices with $1:\textit{N}$ switches and orthogonal waveguide crossing elements into a four-degree input/output silicon-photonics chip, we can now scale up designated multidimensional optical switches needed to design and construct large-scale photonic on-chip interconnects such as photonic intrachip communication networks, central optical add/drop multiplexing transmission equipments, all-optical traffic protection switching nodes, and photonic data centers. Meanwhile, reconfigurability of the proposed multi-degree switches is exploited by manipulating controllable phase shifters under the impact of the thermo-optic effect. Achievements in high-resolution silicon photonics technology allow fabricating reconfigurable and scalable silicon photonic switches with advantages of high compactness, low-loss, low power consumption, and fast switching time. For example, reconfigurable and programmable switches can be attained through the use of external field-driven phase shifters resulting in the considerably low power consumption of milliWats level and a remarkably short switching time of only a few $\mu s$ or *ns*^44–46^.

Once a full-grid PCN with a considerable connection size is created, several questions emerge: (1) how to perform a rapid routing with low cost, and (2) how to infer multiple control in strict conditions and deploy in reality. That is the reason why we propose an optimization algorithm to answer those question as well as address second requirement in our work. Typically, optimization algorithms in electronic switches were primarily developed to resolve contention problems, such as the classic looping algorithm, in which all paths seen are equal. Some optimization algorithms focused on loss and input power dynamic range (IPDR) improvement in Clos network^47^, crosstalk reduction and connection structures arrangement in the dilated Banyan topology^48,49^, controlled-phase balancing optimization in the Benes topology^30^, or mesh networks optimization based on micro-ring resonators with a few of connection ports for wavelength selective switches^50^. However, many of these are unsuitable for optical network environments that have complexity space, dynamic changes. The state of these environments is very stochastic, leading to every heuristic and exhaustive algorithms are failed. This limits switch performance and results in an excessive disturbance in the waveguide, whose factors need to be counted in the photonic routing strategy.

Deep reinforcement learning (DRL) is combination between reinforcement learning (RL)^51^ and deep learning^52^ into a merged solution, allowing agents to make decisions from unstructured input data without manual engineering of the state space. DRL algorithms are able to take in very large inputs and decide what actions to be performed to optimize an objective, thus becoming a strongly developed subfield in the artificial intelligence (AI) and spreading in wide applications such as strategy games^53^, autonomous driving^54^, autonomous control^55^, language processing^56^, mobile robots^57^, IoT security^58^, and communications^59,60^. We, in this investigation, propose Multi-Sample Discovery that utilizes Proximal Policy Optimization (PPO), one of the state-of-the-art on-policy deep reinforcement learning models, for routing in the optical networks^61,62^. Our proposed MSD model is a hybrid Deep Learning model which overcomes PPO’s drawback by exploration ability and inspired by those ideas such as Curiosity-driven Exploration (CDE)^63^ and Hindsight Experience Replay (HER)^64,65^ which is demonstrated better performance by creating denser reward signals from the environment. In MSD, we create an Advisor and Sample Extraction Buffer that are able to auxiliary explore and create multiple efficient samples in PCN^66^, therefore MSD can converge faster to optima. To verify the effectiveness of our proposed method MSD on routing in photonic-chips networks, we design a simulated PCN environment to test it. We let the MSD optimize the transmission, power consumption characteristics and time by providing the optimal routing path. Our experiments comprise of a few comparisons between the performance of our model and several other the state–of–the–art RL models to make sure that MSD is the best fit for PCN. The result shows that MSD significantly outperforms the others in reducing transmission loss, power consumption and routing time. In addition, MSD can improve the speed of the training process while effectively handling noise or sparse signal. Based on the experiment, we see that MSD is easy to apply for routing in optical networks, able to provide optimal strategy in strict condition, adapt to the dynamic environment such as PCN and stable when the number of nodes in the network is large. Such interior-deductive capabilities make the constructed PCN comparable to an all-optical spiking neurosynaptic network^67^.

**Figure 1:**
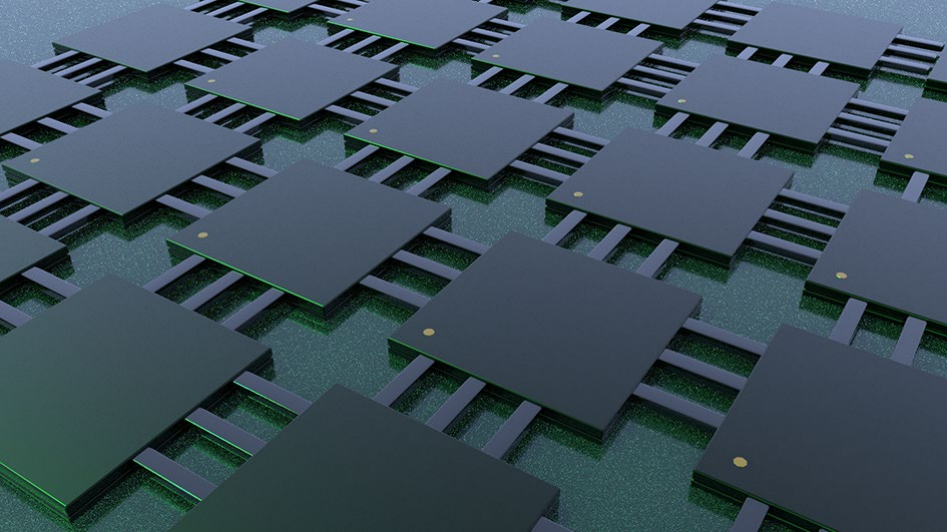
The schematic diagram of a proposed multi-degree optical switch based on the silicon photonic waveguide structure. This is a 2D grid of $M{\times }N$ photonic chips. Each photonic chip is a 3$\times$3$\times$3$\times$3 four-directional structure controlled by thermooptic phase shifters using titanium metallic thin films.

## Multi-degree optical switches

Figure 1 shows the schematic diagram of a proposed full-grid photonic-chip network (PCN) which is constructed by connecting multi-degree optical switches as unit cells in the two-dimensional space following the full-grid topology. Each unit cell is a multi-degree photonic switch based on on-chip silicon waveguide structures, and therefore, the proposed PCN can be monolithically integrated on a standard silicon-on-insulator wafer by manipulating CMOS-compatible fabrication processes. PCN is an $M{\times }N$ rectangle structure comprising *M* rows and *N* columns of photonic chips. Each multi-degree photonic switch enables the operations of the four-degree non-blocking switching connections. Furthermore, the proposed photonic switch can be dynamically programmed corresponding to some arbitrary connection mechanisms from east-west-south-north directions of the 3$\times$3$\times$3$\times$3 structure via the controlling progress at the crossroads by driving thermo-optic phase shifters. In the remaining of this section, we will first describe the components of a photonic chip. We will then explain the control mechanism and report the experiments that confirm the favorable characteristics of our hardware design.

**Figure 2:**
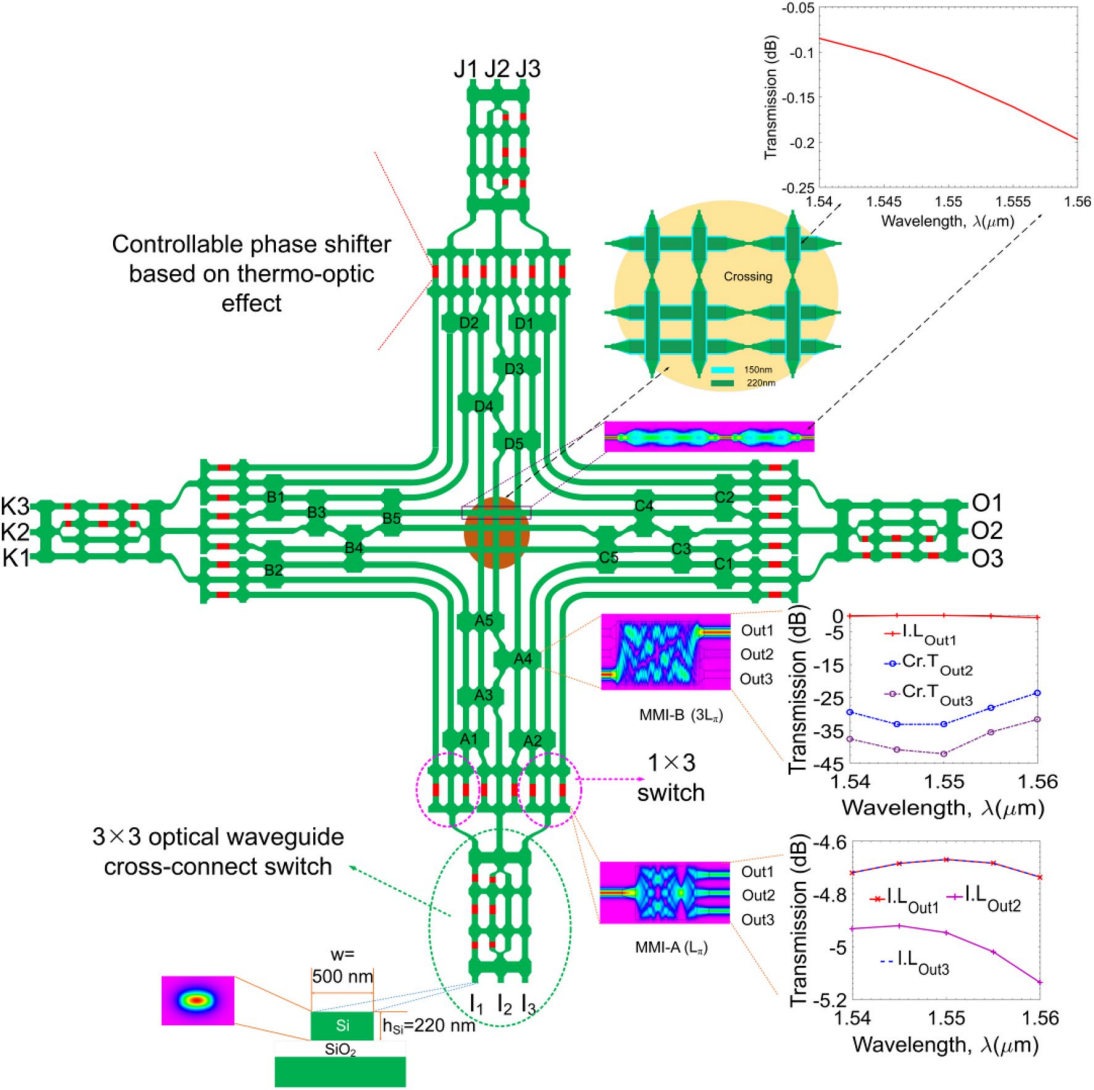
The architecture of our proposed four-degree 3 × 3 × 3 × 3 silicon photonic chip.

### Components of a photonic chip

#### Overall structure of a photonic chip

Figure 2 shows the structure of a four-degree 3 × 3 × 3 × 3 photonic chip based on silicon photonic waveguides. The switch has twelve input/output ports distributed in four groups including North, South, East, and West, where each group has three input/output ports. Inside the switch, a $3{\times }3$ optical waveguide cross-connect switch (OWXC) playing the central role is responsible for connecting and switching optical channels at the waveguide-level. To the best of our knowledge, such a structure has never been suggested before. The three outputs of the OWXC component are connected to three $1{\times }3$ optical switches to divert optical channels in three different directions. The outputs of each $1{\times }3$ switch are connected to the redirection couplers that allow the connection to be redirected in three outbound directions. At the center of the chip is a waveguide crossing mechanism that guides optical waves through the intersections. All major components of a photonic chip are sketched in Fig. 2. The whole structure is constructed on silicon-on-insulator (SOI) material using channel waveguides and patterned by electron beam lithography or deep ultraviolet (DUV)^68,69^. Access waveguides are silicon nanowires with the width $w = 500 \, nm$ for supporting quasi-transverse electric (quasi-TE) single-mode transmission condition at 1550 nm^70^.

#### The OWXC component

The operation of the 3 × 3 OWXC element is based on the multimode interference principle, which allows the self-imaging reproduction periodically^71^. The OWXC has two $3{\times }3$ MMI couplers placed at the input and the output, playing the roles of the optical channel divider and combiner. The multimode length of these MMI couplers is $L_1 = 3L_{\pi }/2$. The OWXC also has two 4 × 4 MMI couplers with the multimode length $L_2 = 3L_{\pi }/4$ in the middle and six controllable phase shifters (marked by the red color in Fig. 2). The central output of the first 3 × 3 MMI coupler and the central input of the second 3 × $\times$3 MMI coupler is the two-mode waveguide acting as a Mach-Zehnder Interferometer (MZI) passing through two 4 × 4 MMI couplers. For switching, controllable phase shifters (PSs) need to be shifted a phase difference of $\pm \pi /2$. The half beat length $L_{\pi }$ of the multimode waveguide is defined by^71^:

1 $$\begin{aligned} L_{\pi } = \frac{\pi }{\beta _0 - \beta _1} = \frac{4 {n_{eff}} {W_e}^2}{3\lambda _0}, \end{aligned}$$

where $\beta _0$ and $\beta _1$ are the propagation constants of the fundamental and the first order modes determined by the following relation^71^:

2 $$\begin{aligned} \beta _\nu \approx k_0n_{eff}-\frac{(\nu +1)^2\pi \lambda _0}{4n_{eff}W_e^2}, \end{aligned}$$

where $\nu$ is the $\nu$th guided mode order into the core waveguide, and $n_{eff}$ is the effective refractive index of the silicon core waveguide layer, obtained by solving the wave-propagation differential equation by using the numerical methods. $\lambda _0$ is the operation wavelength in vacuum, and $W_{e}$ is the effective width of the MMI coupler^71^:

3 $$\begin{aligned} W_{e} = W_{MMI} + \frac{\pi }{\lambda _0} (n_{eff}^2 - n_{c}^2)^\frac{-1}{2} (n_{eff}/n_{c})^{2\sigma }, \end{aligned}$$

where $\sigma = 0$ for TE polarization and $\sigma = 1$ for TM polarization. $W_{MMI}$ is the geometric width of the MMI coupler, and $n_{c}$ is the cladding refractive index ($SiO_2$ material).

#### The 1 $\times$ 3 switches

The proposed multi-degree photonic switch contains three 1 $\times$ 3 switches. Each 1 $\times$ 3 switch consists of a 1 $\times$ 3 MMI coupler at the first section and a 3 $\times$ 3 MMI coupler at the second section. They have the same multimode region with the multimode length $L_3 = L_{\pi }$. Two outermost access arms of two MMI couplers are linked via two controllable phase shifters. Depending on the choice of appropriate phase-difference combinations, either (2$\pi$/3,0), (0, 2$\pi$/3), (− 2$\pi$/3, − 2$\pi$/3), the switch will select the output at the left, middle, or right sides^72^. A numerical simulation of the electric field envelope distribution and the transmission spectral wavelength response characteristics of a 1 $\times$ 3 MMI multimode coupler are shown in the subfigures of Fig. 2. The wavelength-dependent transmission spectral characteristic shows that the coupler acts as a perfect triplet divider with power at the three output ports (Out1, Out2, Out3) approximately 1/3 dividing ratio ($-4.77$ dB) in a 20*nm* bandwidth in the 1550*nm* central wavelength region.

#### MMI couplers

Redirecting waveguides (denoted by the blocks of A1$\div$A5, B1$\div$B5, C1$\div$C5 and D1$\div$D5 in Fig. 2) are $3{\times }3$ MMI couplers with the multimode length $L_4=3L_{\pi }$. These couplers operate on the general interference mechanism (GI-MMI) enabling the input optical field mirrored over the central line of the multimode region and reproducing the optical field at output from the input optical field. Such mechanism makes optical channels in the proposed multi-degree switch redirected flexibly, completely. Images of electric field distribution and wavelength-dependent transmission spectral characteristics are shown in the corresponding insets of Fig. 2. The coupler acts as a near-perfect waveguide crossover with a high transfer rate ($> 96\%$) and a meagre interference ratio not exceeded -25 dB ensuring the switching feature to attain high optical performance in a wide 20*nm* bandwidth, as seen in Fig. 2.

#### Waveguide crossing structure

The central part of the structure is a waveguide crossing structure consisting of three perpendicular silicon nanowires crossing to each other. The operating principle is based on the multimode interference effect at the intersection point of the perpendicular waveguide. The single-mode waveguide crossings are indispensable building and connecting blocks for complex photonic circuits in the system-on-chip. By utilizing fully etched and shallowly etched waveguides and linear tapered waveguides in the MMI coupler region, the waveguide crossing attains ultra-low loss and imbalance^73,74^. The transfer characteristic of the waveguide crossing designed for the proposed multi-degree switch, illustrated in Fig. 2, shows that the transmission loss of this structure is low, only fluctuating from $-0.1$ to $-0.2$ dB in 20 nm wavelength bandwidth.

### Control mechanism

**Figure 3:**
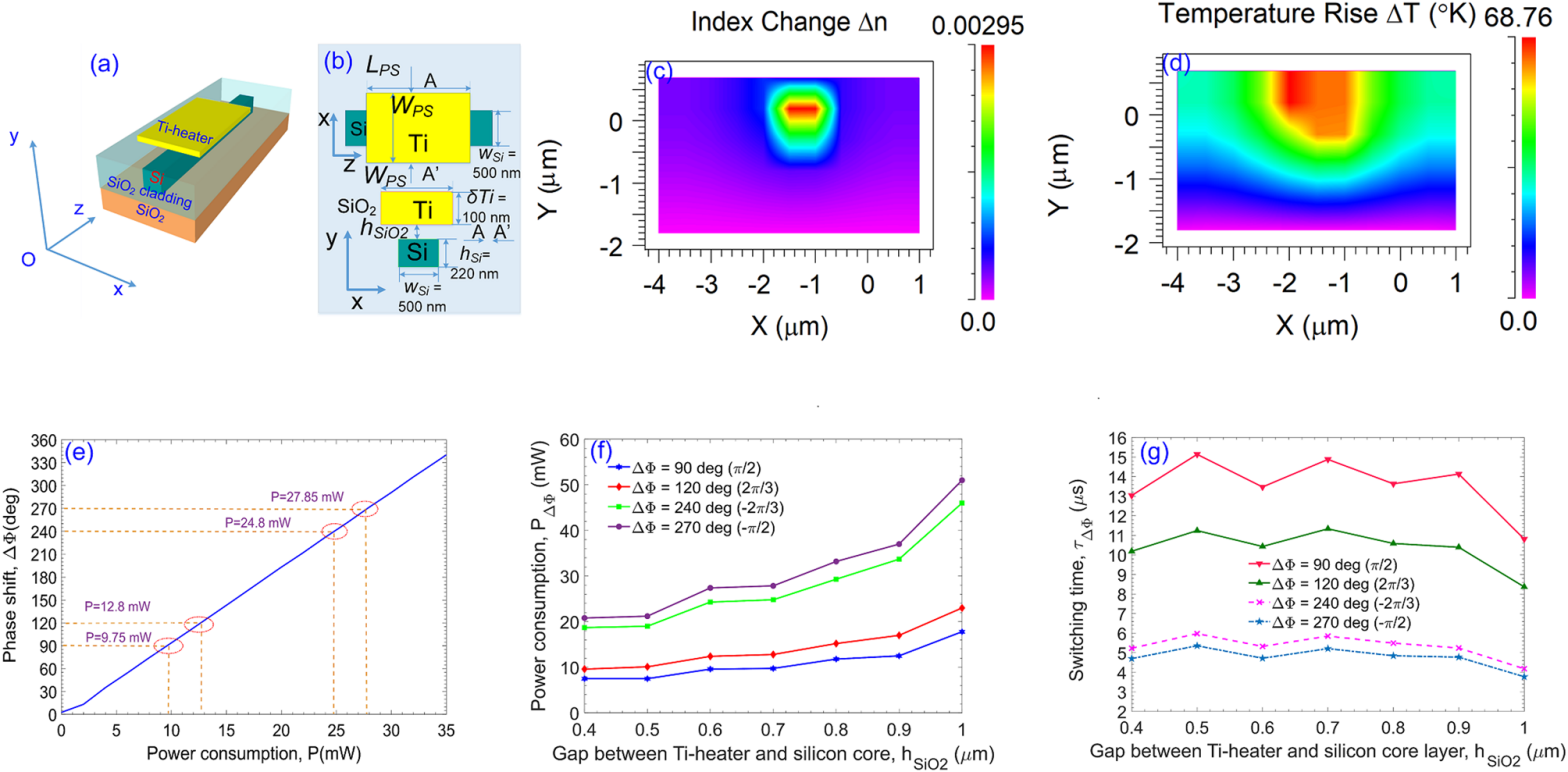
Operation of the TOPS using the metallic Ti-heater **(a)** schematic diagram of the TOPS in the 3D space, **(b)** side-view and cross-view of the TOPS, **(c)** distribution of the index change $\Delta n$ of the silicon core layer via the FEM simulation method, **(c)** distribution of the temperature rise $\Delta T$ simulated by the FEM-based multiphysics tool, **(e)** phase shift $\Delta \phi$ according to electric power consumption supplied to the metallic Ti-heater, **(f)** switching power consumption as a function of the silica gap $h_{SiO_2}$, and **(g)** switching power consumption as a function of the silica gap $h_{SiO_2}$.

Thermo-optic phase shifters controlled by the external voltage source play the critical role for realizing a wide range of integrated photonic applications such as neural networks^75^ and reconfigurable photonic chip^76^ due to ultrafast temporal response, high flexibility, high accuracy, compact size, and CMOS compatibility. For changing the phase angle in silicon photonic waveguides, the thermo-optic effect is applied to modulate the change of the silicon refractive index via the utilization of metallic heaters, such as heater based on the Ti metal thin film, causing the change of the silicon refractive index by the following relation^26,77^:

4 $$\begin{aligned} \Delta \phi = k L_h \Delta n = k {{L}_{{P}_{S}}} \frac{dn}{dT} \Delta T, \end{aligned}$$

where $\Delta$*T* is the change in temperature determined by $\Delta T = T - T_0$; $T_0 = 300 K$ is the room temperature; $dn/dT = 1.84\times 10^{-4}K^{-1}$ is the thermo-optic coefficient of silicon material; $k = 2\pi /\lambda$ is the wavenumber; $\Delta n$ is the total index change of the silicon material; ${{{L}_{P_{S}}}}$ is the length of microheater.

A conventional configuration of a thermo-optic phase shifter has a metallic heater placing on top of the silicon waveguide to induce a phase shift, thanks to the combination of heating and the thermo-optic coefficient of silicon. Typically, metals are high-loss in the third telecom spectrum. Therefore, an upper cladding layer of silica is applied to optically isolate the heater and the silicon waveguide with reasonable spacing distance because a large gap may cause some drawbacks in the performance such as the power consumption and/or the switching speed. In contrast, a small gap may make the phase shifter to suffer a high absorption loss from the plasmonic effect^78^. Microheater-based phase shifters can attain a short switching time on a few $\mu s$ and a relatively low electrical power consumption for several tens of *mW* enabling high benefits for optical performances. This explains why microheaters-based controllable phase shifters are preferable than other kinds such as carrier effect-based phase shifters. The thermo-optic phase shifter (TOPS) utilized in the proposed multi-degree switch composes of a metallic Ti-thin film with the thickness of $\delta T_{i} =100nm$, the Ti-heater width of $W_{PS} = 1\mu m$, which is placed on the top of the silicon core layer an acceptable gap $h_{Si0_2}$ within the range from 700*nm* to 1000*nm*. The active length of the TOPS is initially set $L_{PS}= 200\mu m$ to obtain the optimal value of the product of $P_{\pi }\cdot \tau$ during the operation process of the optical switch^26^. Figure 3a describes the structure in the three-dimensional space, and Fig. 3b describes the details in the side-view and the cross-section of the designed TOPS. Figure 3c,d respectively show the distributions of the index change ($\Delta n$) and the temperature rise ($\Delta T$) in the silicon core layer at the switching state when the electric power is applied to reach a required phase difference of $\pi$ radian via the use of the Finite Element Method (FEM) simulation method. The required temperature increases about 68.76 K at the metallic heater for reaching the phase difference of $\pi$ radian. Figure 3e presents the shifted phase angle ($\Delta \phi$) as a linear function of the electric power consumption ($P_{\Delta \phi }$) under the influence of the TOPS which is simulated by using the FEM-based multi-physics tool. The needed powers to reach the required phase angles of $\pi /2$ ($90^{\circ }$), $2\pi /3$ ($120^{\circ }$), − $2\pi /3$ ($240^{\circ }$) and − $\pi /2$ ($270^{\circ }$) measured from simulation data are corresponding to 9.75 mW, 12.8 mW, 24.8 mW and 27.85 mW, respectively. Figure 3f,g correspondingly illustrate simulation results of the electric switching power consumption and the switching time as functions of the isolation gap between the silicon core layer and the metallic Ti-heater $h_{SiO_2}$. Here, the switching power consumption ($P_{\Delta \phi }$) is a specific parameter representing the power efficiency for reaching a phase shift of $\Delta \phi$, which can be determined via the utilization of a modified two-dimensional treatment of the heat transformation model on the lateral spreading as follows^79^:

5 $$\begin{aligned} P_{\Delta \phi } = \frac{\lambda K_{SiO_2} \left( \frac{W_{PS}}{h_{SiO_2}} + 0.88 \right) }{\left| \frac{\partial n}{\partial T}\right| _{\Delta \phi }}, \end{aligned}$$

where $K_{SiO_2}=1.4 W/(m.K)$ is the thermal conductivity of SiO2, $\lambda$ is the operation wavelength, and $W_{PS}$ is the width of the Ti-metal film on the lateral direction, $\left| \frac{\partial n}{\partial T}\right| _{\Delta \phi }$ is the difference of ${\partial n}$ on the difference of ${\partial T}$ for reaching the required phase change of $\Delta \phi$. Whereas, the switching time characterized by the response time of the TOPS has a direct relation to the cut-off frequency by $\tau = \frac{1}{e.f_{cut-off}}$, where $e\approx 2.718281828459$ is the natural logarithm constant. In which, the cut-off frequency is directly related to the switching power as follows^80^:

6 $$\begin{aligned} f_{cut-off} = \frac{P_n}{\pi \lambda \rho _{SiO_2}C_{SiO_2}A} \left| \frac{\partial n}{\partial T}\right| _{\Delta \phi } \end{aligned}$$

where $\rho _{SiO_2} =2.203\ {\mathrm{g/cm}^3}$ is the density of silica, $C_{SiO_2}=0.703\ \mathrm{J/gK}$ is the specific heat capacity, and *A* denotes the effectively heated cross-section area relating to the geometry parameters of the TO phase shifter.

To supply the heat source for creating various temperature change levels in the switching operation, each individual microheater needs to be connected to a individual pulsed-voltage source. Pulsed-voltage sources have an ideal configuration of 5V peak to peak at a repetition rate of 12 kHz superimposed with a DC (direct current) biasing voltage across two contact points at the beginning and ending sides along the length direction ${{L}_{{P}_{S}}}$ of the microheater with an excellent electronic conducting quality of the wire bonding pads by using the noble metals^81^.

### Power transfer and validation experiments

**Figure 4:**
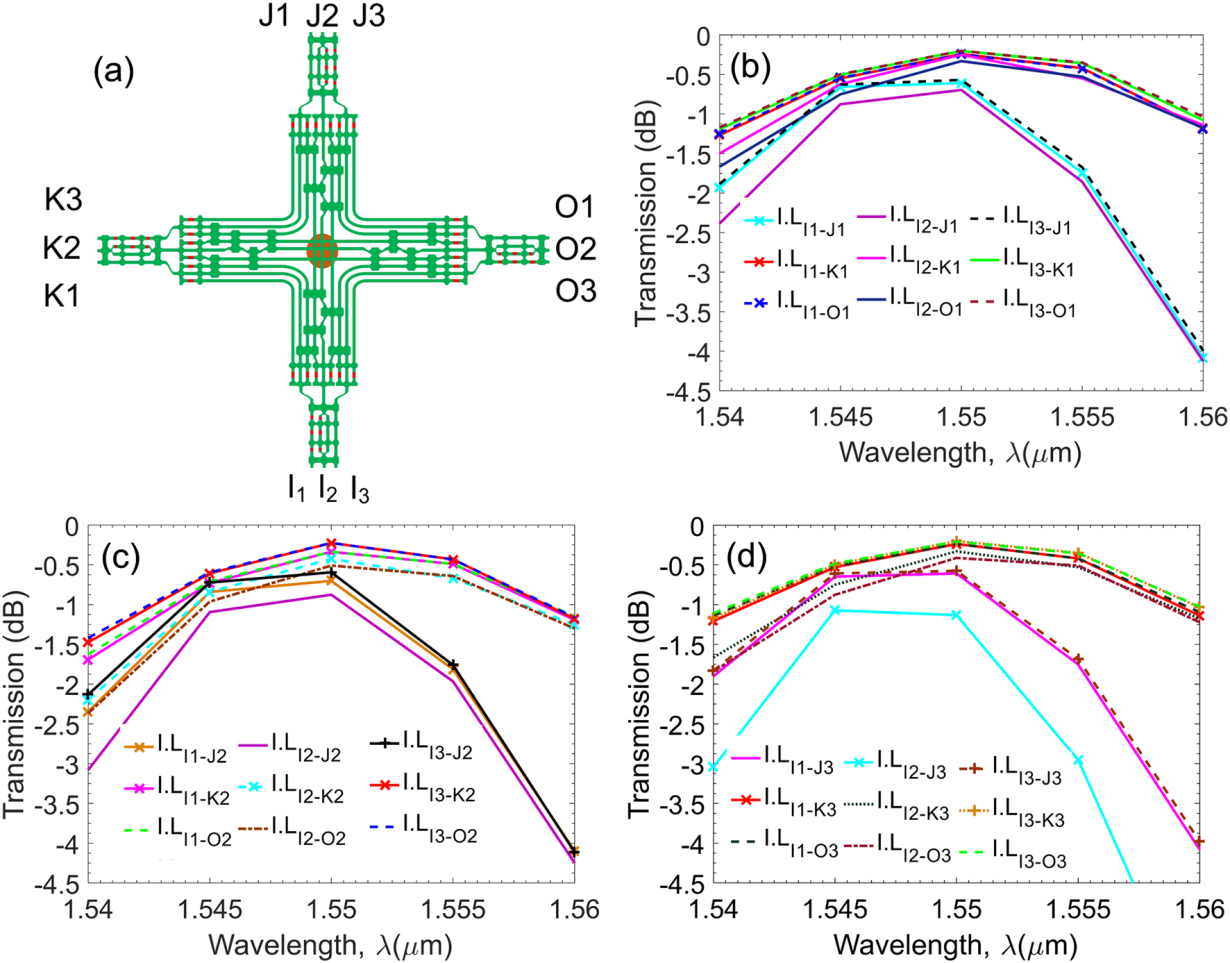
Simulation results of I.L via the numerial simultion method for 27 specific switching states as functions of the wavelength in 20-nm bandwidth when crosstalk is maintained lower than $-20$ dB: **(a)** conceptual diagram with four groups of input/output ports I,J,K,O; **(b)** switching states from channels in the I group to the first channels in three remain groups; **(c)** switching states from channels in the I group to the second channels in three remaining groups; **(d)** switching states from channels in the I group to the third channels in three remain groups.

**Figure 5:**
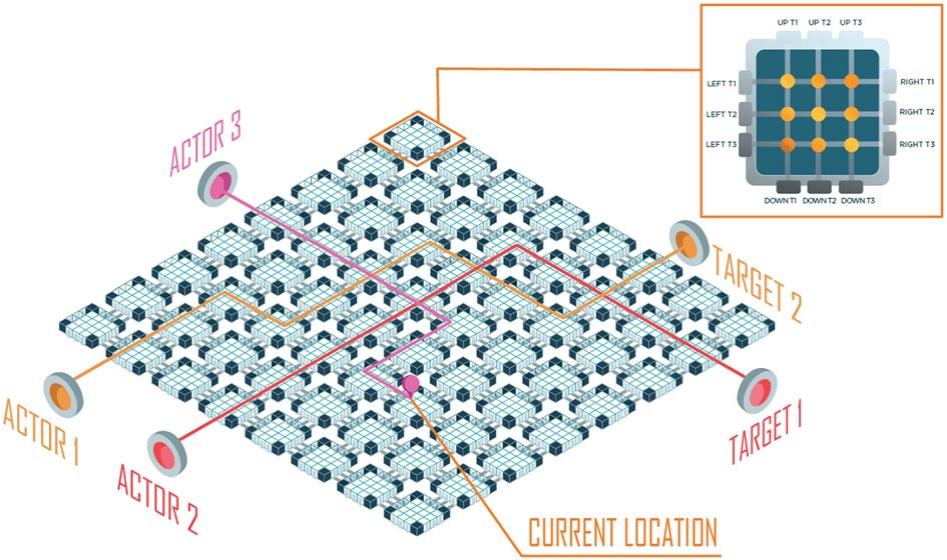
A typical routing problem in a Photonic Chip Network (PCN), where we need to route information between multiple input-output pairs simultaneously.

In general, a photonic device should be low transmission loss in a wide wavelength bandwidth. Especially, for the proposed photonic chip network designed to be a full-grid and large-scale size chip network at the waveguide-capacity level, it should assure a high optical signal-to-noise ratio from any input ports to any output ports at a specific wavelength in the third telecom window (near 1550-nm region). This feature is demonstrated by using the transfer matrix relations. Optical power transfer functions are essential to verify first in the multi-degree $M{\times }N$ switch matrix for each switching state, i.e., sweeping all switch cells is straightforward, cross, left-hand turning, and right-hand turning states requiring a full optical power transfer characteristic map. Consider an input port *i* and an output port *j* at any directions (East, West, North South). Let $\rho _i$ is the injected power level at the input port *i*, and $\rho _{ij}$ the power level at the output. Let $\sigma _{ijk}$ be the leakage power to another port *k*, where $k \ne j$. We introduce most important parameters relating to optical performances of light paths in an optical switch to be the insertion loss $\chi _{ij}$ and the crosstalk ratio $K_{ijk}$, which are defined as follows^82^:

7 $$\begin{aligned}&\chi _{ij} = \rho _{ij} / \rho _{ijk} \end{aligned}$$

8 $$\begin{aligned}&K_{ijk} = \sigma _{ijk} / \rho _{ij}. \end{aligned}$$

For each switching state of a pair from input port *i* to output port *j*, the optical power transmission function is a bijection, meaning $\varepsilon _{ij} = \varepsilon _{ji}$. The aggregated crosstalk power at full switch load to an output port *k* is the total undesired powers leaked to *k* from all *i* to *j* transmission paths, which can be expressed as:

9 $$\begin{aligned} \mu _{k} = \sum _{i}\sum _{j\ne k} K_{ijk}. \end{aligned}$$

The extinction ratio for output *k* can be written as:

10 $$\begin{aligned} \xi = \mu _{k} / \rho _{ij}. \end{aligned}$$

The insertion loss and crosstalk in *dB* can be expressed by:

11 $$\begin{aligned} I.L = 10\log _{10}(\chi_{ij}), \end{aligned}$$

12 $$\begin{aligned} Cr.T = 10\log _{10}(\mu_k), \end{aligned}$$

Figure 4 shows the data obtained by a numerical simulation for 27 specific switching states for a variety of input-output pairs from all groups I, O, J, K. The figure shows the values of the insertion loss when the wavelength range is varied from $1.540 \upmu$ m to $1.560 \upmu$ m. As can be seen in these subfigures, the insertion loss I.Ls do not exceed 4.5dB while keeping crosstalk under −20 dB for all cases of switching states in the experimented 20 nm wavelength bandwidth. By observing subfigures in Fig. 4 directly, one can see that, transmission characteristics curves of insertion loss at the wavelength smaller than 1550 nm are less variant. All characteristic curves gradually increase in 10-nm bandwidth from 1540 nm to 1550 nm and they are relatively close in the wavelength range from 1545 nm to 1550 nm. Besides, almost insertion losses attain the optimal values at the central wavelength of 1550 nm agreeing to the aimed targets because all of discrete elements are optimally designed on aspect of insertion loss at the central wavelength of 1550 nm. Furthermore, all characteristic curves of insertion loss decrease when the operation wavelength is larger than 1550 nm. However, characteristic curves are split into two major groups. The first group composes of characteristic curves of connection paths coming from waveguide channels in the adjacent input/output ports, for example, the connection paths from $I_i$ to $K_j$ or $O_j$ (*i,j*=1,2,3) channels. The second group includes characteristic curves of connection paths coming from waveguide channels in the vertical and horizontal directions after passing through the waveguide crossing region. Wavelength spectra responses of insertion loss transmissions in the first group gradually reduce like the wavelength spectra response of a silicon multimode waveguide resulting from the loss profile of silicon crystal and the unpreservable phase-matching condition. Among these connection paths, the transmission property of the cross channels is better than the transmission property of the central-straightforward channels, for instance, the insertion loss transmissions of $I_3$-$K_1$ and $I_3$-$K_2$ connections are better than the insertion loss of the $I_2$-$K_2$ connection. This is because transmission of outer arms in the 3-dB MMI coupler followed by the general interference regime is better than the transmission of the central inner arm in the MMI coupler agreed by the symmetric interference regime, as seen in Fig. 2. On the contrary, wavelength spectra responses of insertion loss transmissions in the second group are always smaller than that of the first group due to considerable insertion loss of the connection paths when surpassing the waveguide crossing sections. In addition, since the operation wavelength is larger than 1550 nm, transmission curves of connection paths in the second group dramatically fall down because, beside the unpreservable phase-matching condition, the transmission characteristics of connection paths must suffer a remarkably accumulative loss from the waveguide crossing region, as seen in the subset figure exhibiting the transmission property of the waveguide-crossing element. Therefore, the 3-dB wavelength bandwidth responses of straightforward cross-connection paths are narrower than the 3-dB wavelength bandwidth responses of adjacent cross-connection paths in a multi-degree optical switch. Furthermore, one can see that insertion loss transmission characteristics attain correspondingly to the best values of about 0.25 dB and the worst values of about 1.1 dB at the central wavelength of 1550 nm, respectively. However, such low insertion loss and crosstalk levels still have a relatively wide bandwidth of 20-nm, demonstrating the excellent performance of the proposed multi-degree switch.

**Figure 6:**
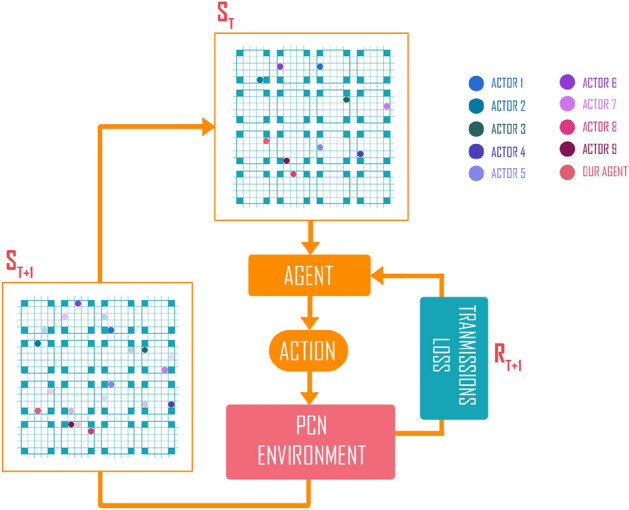
Markov Decision Process in PCN environment. $S_T$ and $S_{T+1}$ are the state of the environment after the agent takes an action. $R_{T+1}$ is the reward, obtained based on the transmission loss.

## Routing for photonic chip network (PCN)

In the previous section, we have described the components of a photonic chip and demonstrated the possibility of transferring the power from one input port to an output port of the same chip with very low insertion loss and cross talk. In this section, we will describe our proposed routing policy for a full-blown optical switch, which is a Photonic Chip Network (PCN) of $N{\times }M$ photonic chips, where we need to route information from multiple inputs to multiple outputs simultaneously and dynamically.

The routing task on a PCN can be formulated as traffic routing problem, where the PCN is considered as a road network with four directions of size $3M{\times }3N{\times }3M{\times }3N$. Under this formulation, transmitting a signal from an input to an output port is equivalent to directing a traffic agent towards its designated destination, as illustrated in Fig. 5. At each time step, there might be multiple traffic agents in the road network, and our task is to specify the next way point for each agent to advance toward its destination. Ideally, we want to find the shortest path for each road agent, but this is a challenging task given the need to optimize for transmission criteria and avoid collision. Furthermore, we need an efficient algorithm, especially when frequent recalculation is unavoidable due to the dynamics of the network.

Network routing is a well-studied problem but many existing algorithms are unsuitable for a switch network. Traditional planning algorithms such as A*^83^ and D*^84^ are too slow for dynamic environments. Simpler heuristics such as Hill Climbing^85^ are faster, but the provided routing path for each traffic agent can be far from optimal due to the greedy action that does not account for the long-term consequence. However, finding the optimal routing path is a sequential decision process, where the next way point of the agent will affect the state of the entire traffic network and the future course of actions. This problem is very much amenable to Reinforcement Learning (RL)^84,86^, and we propose to use RL to learn a routing policy.

In the remaining of this section, we will first describe the main components of our RL formulation, including the state representation, the reward function, and the action space. We will then describe a novel algorithm to learn the RL policy called Multi-Sample Discovery PPO (MSD). MSD is based on Proximal Policy Optimization^87^, but it is particularly designed for switch network environments.

**Figure 7:**
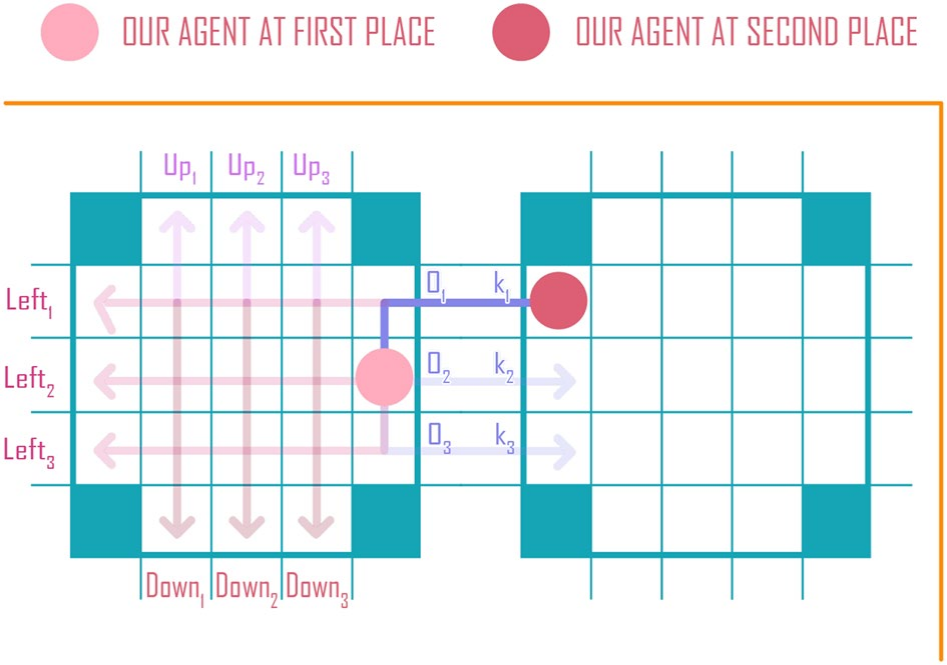
Possible actions of an agent in a PCN environment. At each location, the agent can take 12 possible actions. Depending on the action, the agent can remain on the same photonic chip or move to an adjacent one.

**Figure 8:**
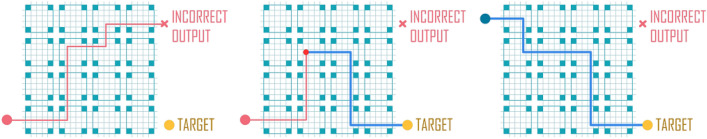
Learning the episode that corresponds to a failed routing. Left: a failed route that does not lead to the target destination. Middle: a new successful route obtaining by replacing the second part of the failed route with that of the successful one. Right: a successful route from the Sample Extraction Buffer (SEB).

### Reinforcement learning formulation

We now describe the main components of our reinforcement learning formulation, which are the state representation, the action space, and the reward function.

#### State representation

Given a PCN with $M{\times }N$ photonic chips, we will use a three-dimensional tensor of size $3M{\times }3N{\times }2$ to represent the state of the PCN at each time step. This state representation encodes the current locations of all traffic agents in the network, the cumulative transmission loss of each agent, and also the destination of each agent. This state representation is obtained as follows. First, for each photonic chip *c* of the PCN structure, we will construct two $5{\times }5$ matrices $L^{c}$ and $D^c$ to represent the state of the chip. Without counting the corner entries, each of these two $5{\times }5$ matrices has exactly twelve entries along the outer edges of the matrix, and each entry corresponds to a specific port of the photonic chip. Let $L^c_i$ and $D^c_i$ denote the entries of $L^c$ and $D^c$ that correspond to the $i^{th}$ port of the chip ($1\le i \le 12)$. If there is an agent *k* at port *i*, we will set $L^c_i$ to *k* and $D^c_i$ to the cumulative transmission loss of agent *k*. If the destination of an agent $k'$ is at port *i*, we set $D^c_i$ to $k'$. Thus, we use $D^c$ to encode both the transmission losses and the destinations of agents. This is possible because an agent should not be at the destination of another agent. **Second**, the matrices $L^c$ and $D^c$ can be stacked to create a $5{\times }5{\times }2$ tensor to represent the state of the photonic chip *c*. **Third**, we spatially concatenate the state representations of all photonic chips together to create a $5M{\times }5N{\times }2$ tensor, as illustrated by $S_T$ and $S_{T+1}$ in Figure 6. Finally, we resize this tensor to $3M{\times }3N{\times }2$, and it is used as the state representation for the PCN.

#### Action space

A RL agent in a PCN has 12 possible actions at each time step: Left1, Left2, Left3, Right1, Right2, Right3, Up1, Up2, Up3, Down1, Down2, Down3, corresponding to four directions and three possible ports per direction. Depending on the action, the agent can remain on the same chip or move to an adjacent chip. For example, consider a specific agent at port *O*2 (Fig. 7). This agent will remain in the same chip if it takes any Up, Down, Left action. This agent will move to either port *K*1, *K*2 or *K*3 of the chip on the right side of the current chip, if the agent takes any of the actions: Right1, Right2, or Right3. If the agent is already at the right most edge of the PCN (i.e., no chip on the right side of the current chip), the agent will remain at the same location if it takes a Right action.

#### Reward function

The reward of an agent after each action is set to be the negative of the transmission loss. We first run a PCN simulator to compute the transmission loss corresponding to the photonic chip’s input and output. When the agent takes action at time *t*, the agent receives the reward $R_t = - IL$, where *IL* is the insertion loss defined in Eq. (11). By learning a policy to maximize the sum of rewards $\Im= \sum _{t=0}^T R_t$, we will obtain a routing policy that minimizes the total transmission loss.

**Figure 9:**
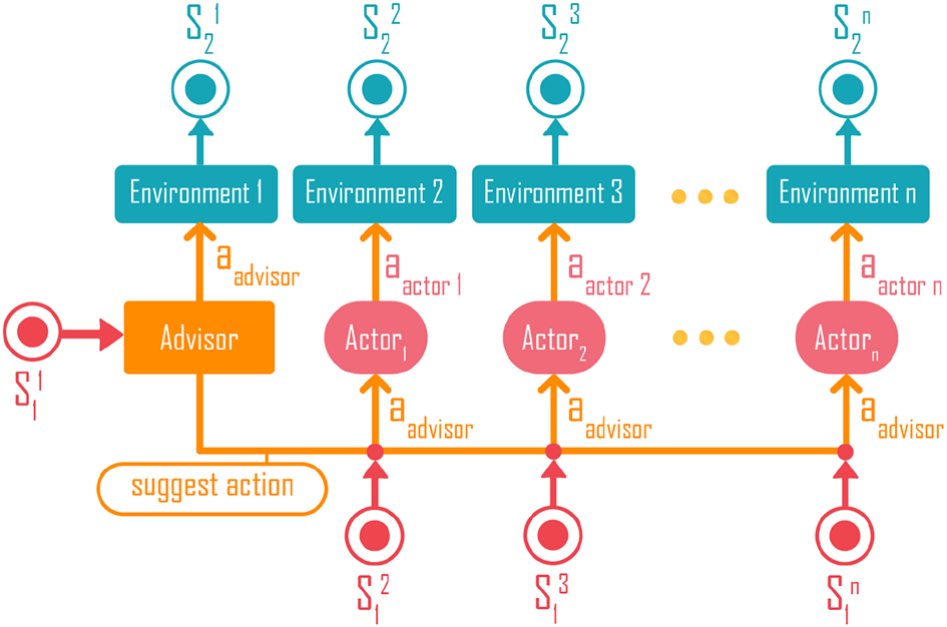
MSD includes a novel component called the advisor. The advisor is a special actor, trained with data from the Sample Extraction Buffer (SEB). The advisor provides suggestion to the actors (there are multiple actors exploring the environment at the same time).

### Learning the reinforcement learning policy

To learn the optimal routing policy, we use Proximal Policy Optimization (PPO)^87^, a state-of-the-art reinforcement learning algorithm. The PPO is a type of policy gradient algorithm, which is an iterative optimization procedure where the parameters of the policy are updated based on the gradient of a loss function defined based on the agent’s interaction with the environment and the rewards it receives. PPO is an on-policy algorithm, meaning that the agent uses its own policy to interact with the environment to generate interaction data sequences for optimizing the policy. Each interaction data sequence is called a learning episode, and in our case, it is a sequence of state-action-reward triplets as the agent is routed by the current policy from an input port to a desired output port. A learning episode can be a successful or unsuccessful routing, depending on whether the agent reaches the designated destination. The PPO is a robust and easy to use, but it is not data efficient because each learning episode is used only once for training the policy. Furthermore, the PPO might be trapped in a vicious cycle of bad policy and bad data, where the bad policy does not generate useful data to improve the policy. To address these problems, we develop here a novel algorithm called Multi-Sample Discovery PPO (MSD), which extends the PPO by maintaining a Sample Extraction Buffer (SEB) that stores learning episodes that correspond to successful routing. During training, MSD will first use its policy to generate a learning episode. If this learning episode is a successful routing, MSD will not be different from the PPO; it will use the learning episode to update the parameters of the actor and the critic functions, which are the main components of the PPO algorithm^87^. However, if the learning episode is a failed routing attempt, MSD will effectively find in SEB a successful route that shares a common node (in the traffic network) with the failed routing attempt. The part of the failed route after the common node is then replaced by that part of the successful one to create an updated learning episode that corresponds to a successful route, as illustrated in Fig. 8.

In MSD, we also introduce a novel component called the advisor function, which is maintained in addition to the actor function of the normal PPO algorithm. The advisor is essentially a special actor that is trained based on the samples provided by the SEB when bad samples are encountered. The role of the advisor is to provide suggestion to the multiple asynchronous actors that are deployed to explore the environment in parallel, as illustrated in Fig. 9.

Let $\pi _{\theta _{actor}}$ denote the policy function of the actors with $\theta _{actor}$ being the vector of parameters of the policy function. At each training iteration, $\theta _{actor}$ is updated to $\theta$ that maximizes the following objective^87^:

13 $$\begin{aligned}&\theta _{actor} = \nonumber \\&\mathop {\text {argmax}}_{\theta } \hat{\mathbb {E}}_{t}\left[ \min \left( r_{t}(\theta ) {\hat{A}}_{t}, {\text {clip}}\left( r_{t}(\theta ), 1-\epsilon , 1+\epsilon \right) {\hat{A}}_{t}\right) \right] , \end{aligned}$$

where the expectation $\hat{\mathbb {E}}_{t}$ indicates the empirical average over a finite batch of samples, ${\hat{A}}_t$ is an estimator of the advantage function at timestep *t*, $r_t(\theta )$ is the probability ratio between the sought-after policy and the old policy, and ${\text {clip}}\left( r_{t}(\theta ), 1-\epsilon , 1+\epsilon \right)$ is the clipping function that clips $r_t(\theta )$ between $1-\epsilon$ and $1 + \epsilon$.

In PPO^87^, the probability ratio is

14 $$\begin{aligned} r_t(\theta ) = \frac{\pi _{\theta }(a_t|s_t)}{\pi _{\theta _{actor}^{old}}(a_t|s_t)}, \end{aligned}$$

where $\theta _{actor}^{old}$ is the vector of policy parameters before the update, and $a_t$ is the action taken by the actor.

In MSD, there is an advisor and the advisor suggests which action to perform for each actor. The usefulness of the advisor’s suggestion is measured based on the ratio between the average total sum of rewards of the advisor and the actors (averaging over ${\mathcal {K}}$ learning episodes):

15 $$\begin{aligned} {\mathcal {H}}_p =  {\overline{{\Im}}}_{advisor}/{\overline{{\Im}}}_{actor}. \end{aligned}$$

If ${\mathcal {H}}_p \le 1$, the advisor function is not better than the actor function, so the actors act based on their own policy. In other words, the action $a_t$ is sampled from the policy function of the actors, i.e., $a_t \sim \pi _{\theta _{actor}^{old}}(a_t|s_t)$. The probability ratio $r_t(\theta )$ is set based on Eq. (14).

If ${\mathcal {H}}_p > 1$, the advisor function is better than the actor function, and the actors follow the actions suggested by the advisor. The action $a_t$ taken by an actor is sampled from the advisor function: $a_t \sim \pi _{\theta _{advisor}}(a_t|s_t)$. The probability ratio is:

16 $$\begin{aligned} r_t(\theta ) = \frac{\pi _{\theta }(a_t|s_t)}{\pi _{\theta _{advisor}}(a_t|s_t)}. \end{aligned}$$

When the performance of the actor is worse than some expected value, the advisor will revert the actor into the balanced state. In the photonic chips network environment, the reward *R* is simulated from Photonic simulation, while the advisor, the actor, the critic are multi-layer perceptron networks with Exponential Linear Unit activation functions. 
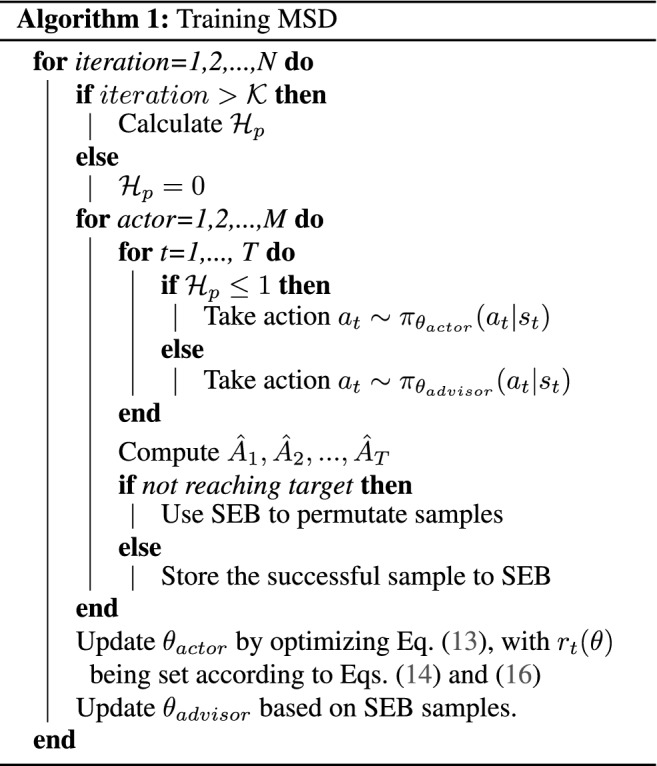


## Experimental evaluation

### Data and environment

We perform experiments on simulated data generated by the photonic component simulator tool with size of 16Mb in plain text. The data composes of transmissions loss from an input port (i) to an output port (j) on a photonic chip with various wavelengths. The example of data structure from an input port to an output port is presented in Table 1. The reward $R_{ij}$ for moving from the input port (i) to the output port (j) is based on the transmision loss and total cross-talk, as specified in Eqs. (11) and (12). From Table 1, the reward can be calculated as:

17 $$\begin{aligned} R_{ij} = -\log _{10} (Output) - \sum _{k=1}^4 Loss(k). \end{aligned}$$

**Table 1:** Example of data generated by the photonic component simulator. $\lambda$ is the wavelength of one specified optical signal. The Output column is output transmission, and Loss1, Loss2, and Loss3 represent the crosstalk of four directions of the agent going through.

$\lambda$	Output	Loss(1)	Loss(2)	Loss(3)
1.53	6.27E−04	7.01E−05	7.36E−03	6.42E−04
1.54	4.04E−01	3.50E−03	4.20E−02	1.45E−02
1.55	6.38E−01	6.49E−03	2.74E−02	3.64E−02
1.56	4.54E−01	2.35E−02	3.14E−03	3.13E−03

In the experiment, the official size of PCN is investigated as 36$\times$36$\times$36$\times$36. The number of actors available in the PCN is equivalent to the number of input-output pairs in PCN (36 actors). In order for the agent to adapt to the new route when the physical failure ports occur on the network, we generate erroneous nodes corresponding to these physical failure ports in a range from 16 to 32 by randomly choosing the available nodes in the PCN environment. In that case, if the agent enters an error node, we will add some penalty to the $R_{ij}$ by doubling the transmission loss from port i to port j.

**Figure 10:**
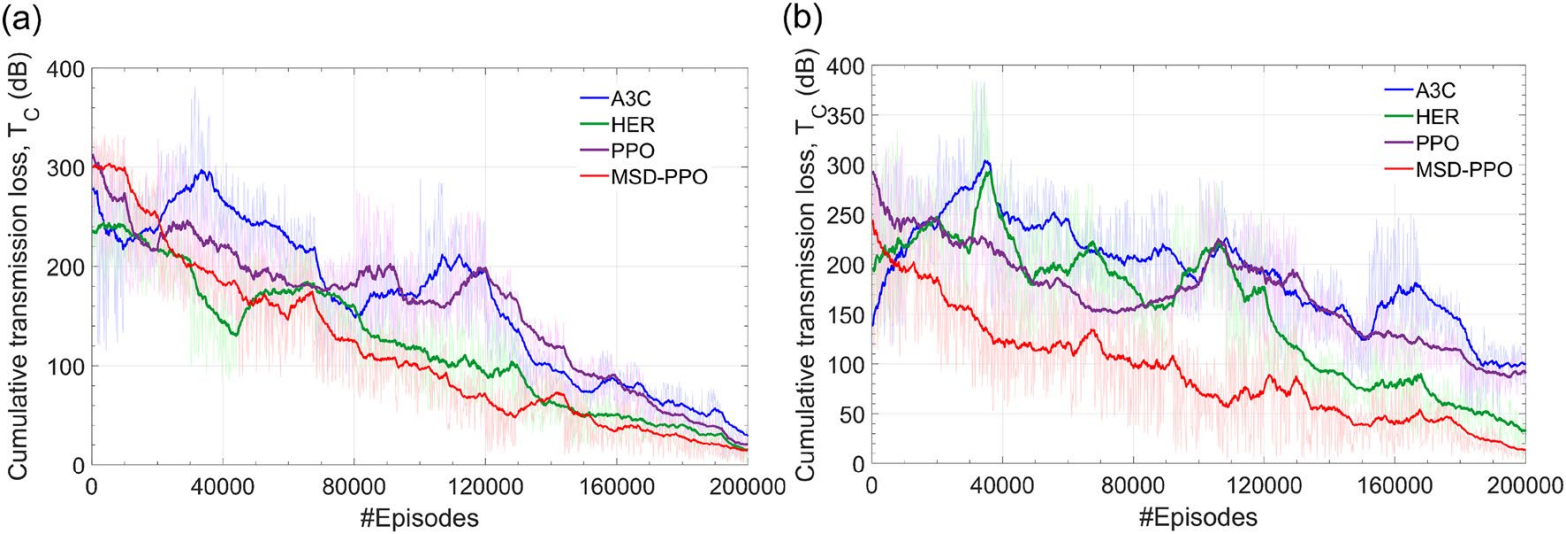
The smoothed cumulative transmission losses of MSD and several reinforcement learning algorithms, including A3C, HER, PPO, when the number of learning episodes increases from 0 to 200K: **(a)** results for environments without error nodes, and **(b)** results for environments with error nodes.

**Figure 11:**
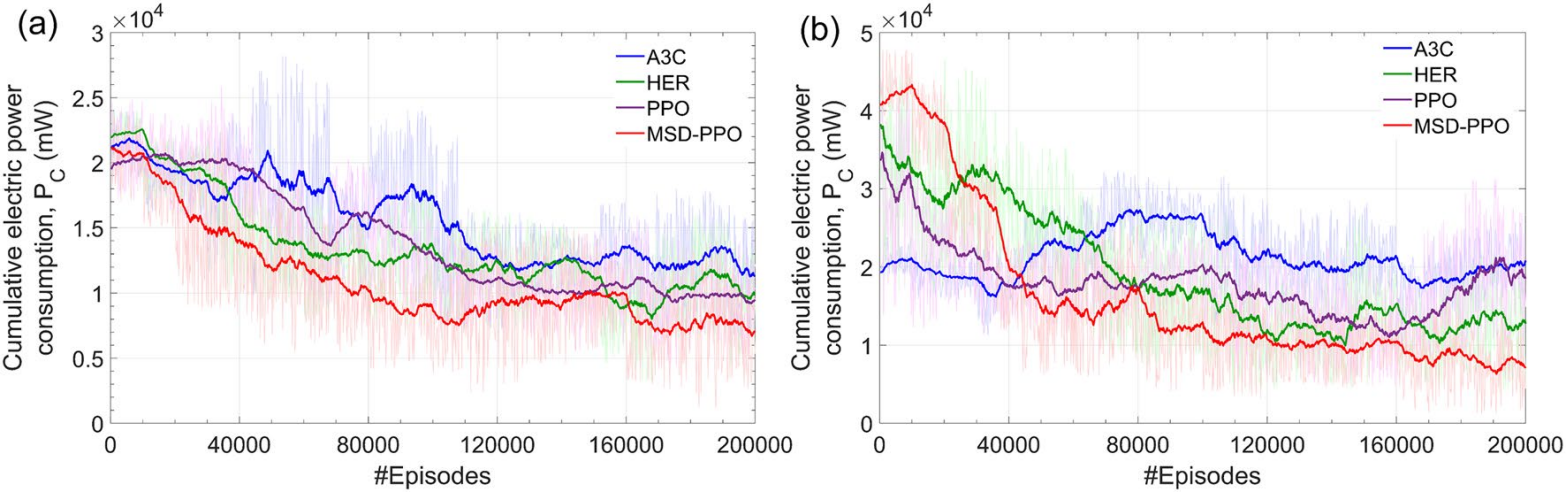
The smoothed cumulative electric power consumption of MSD and several ther reinforcement learning algorithms A3C, HER, PPO when the number of learning episodes increases from 0 to 200K. **(a)** Results for environments without erroneous nodes. **(b)** Results for environments with erroneous nodes.

**Figure 12:**
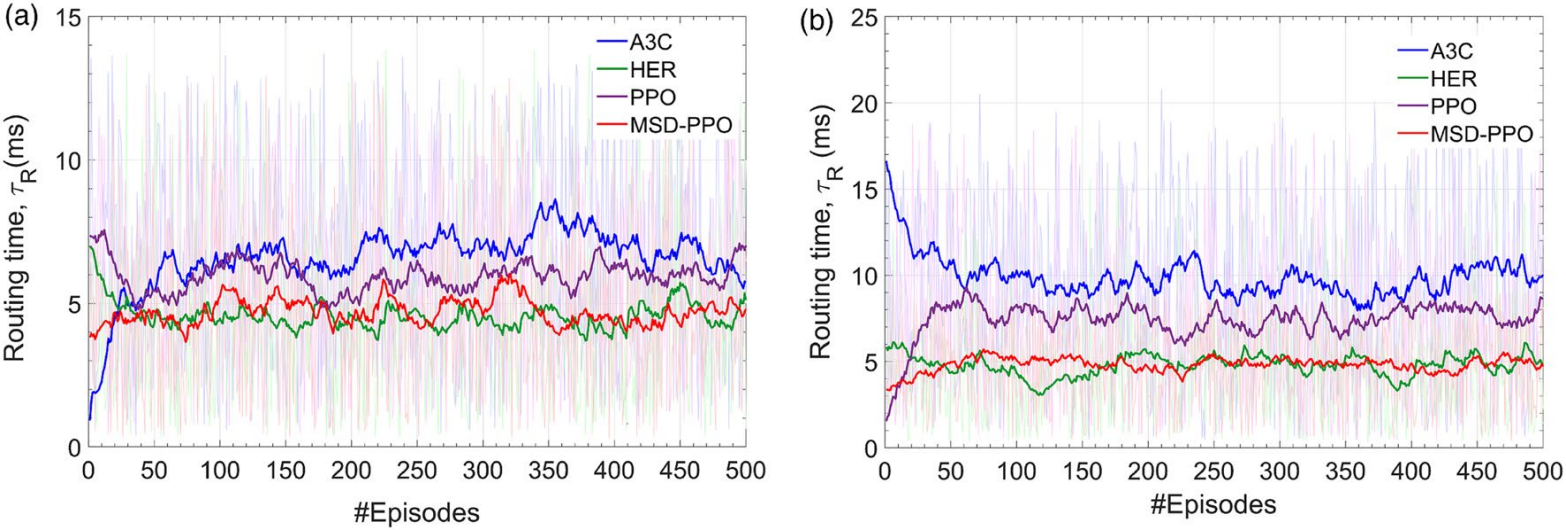
The smoothed total routing time of MSD and several reinforcement learning algorithms A3C, HER, PPO in 500 episode after finish the training process. **(a)** Results for environments without erroneous nodes. **(b)** Results for environments with erroneous nodes.

### Comparison algorithms and metrics

We compare the performance of MSD with several other state-of-the-art reinforcement learning algorithms: PPO^87^, A3C^88^, and HER^89,90^. We use several performance metrics, including: cumulative transmission loss, cumulative power consumption, and routing time.

### Parameter settings

The model parameters of MSD are fine-tuned though a pilot experiment using a subset of the dataset, which provides the optimal values as follows. Co-Efficient Entropy is set to 0.02, while Co-Efficient is 0.05. The Clipping value $\epsilon$ is set to 0.2 and $\lambda$ is set to 0.97. For the network architecture, the number of dense layers is 3 with the number of units being 512. The convolutional layer is made to be false, and ${\mathcal {K}}$ is set to 50. The SEB memory is 100K samples, and the number of permutations on SEB is 50.

### Results and analysis

#### Transmission loss

Figure 10 shows the cumulative transmission losses of MSD and several other reinforcement learning algorithms on the variation of the number of training episodes. As can be seen, MSD converges faster than the other algorithms and attains a routing policy with the smallest level of the cumulative transmission loss. The cumulated average losses for randomly routed optical paths are altered from 4 dB to 15 dB when the MSD model is trained successfully even the M$\times$N sizes are significantly large up to $36\times 36$ for both environments with and without erroneous nodes. These span losses are within acceptable margins for the operating limit of an optical signal transmitting–receiving system, thus indicating an excellent routing quality of MSD when compared to other reinforcement learning algorithms, which have been investigated in simultaneous experiments. For example, when the erroneous nodes are random in the dynamic range from 16 to 32, A3C and PPO can spend average losses up to 90 dB and 100 dB on routing the optical paths, as seen in Fig. 10. Such attenuations are so dreadful that none of the photonic systems can operate in that condition. As a consequence, MSD-PPO can help the photonic network save amount of power margins to enlarge the network size as well as the propagation distance while ensuring the excellent transmission quality in terms of the bit error rate and optical signal to noise ratio in a defined bandwidth. This means MSD-PPO can attain the largest optical spectrum when compared to other reinforcement learning-based algorithms. Therefore, our PCN can support the routing operation for high-load traffic channels.

#### Power consumption

Figure 11 compares the power consumption levels of four reinforcement learning algorithms on chip networks with and without erroneous nodes. In both situations, the MSD leads to a final routing policy with the lowest power consumption that is not exceeded 4 W. After finishing a successful deep-learning process, MSD can provide a sufficient power consumption under 10 W even though the erroneous number can be relatively large, up to 32. For the large PCN size as experienced, this level is significantly economical. This help save energy and improve the lifetime of the constructed PCN.

#### Routing time

Figure 12 compares the routing time of MSD with PPO, A3C, and HER. As can be seen, MSD enables the routing time as approximately as HER. MSD outperforms both PPO and A3C with an awe-inspiring routing time of about 5 ms for both cases with erroneous and without erroneous nodes in a large scale of the experienced PCN size. This result demonstrates that MSD is capable of implementing real-time processing tasks. As must be recalled, MSD is an extension of PPO, and the performance advantage of MSD can be reasoned from the remarkable contribution of the Sample Extraction Buffer. With the excellent value of the routing time, PCN can reroute a optical channel instantly assuring the continuous information connection without disconnection.

**Figure 13:**
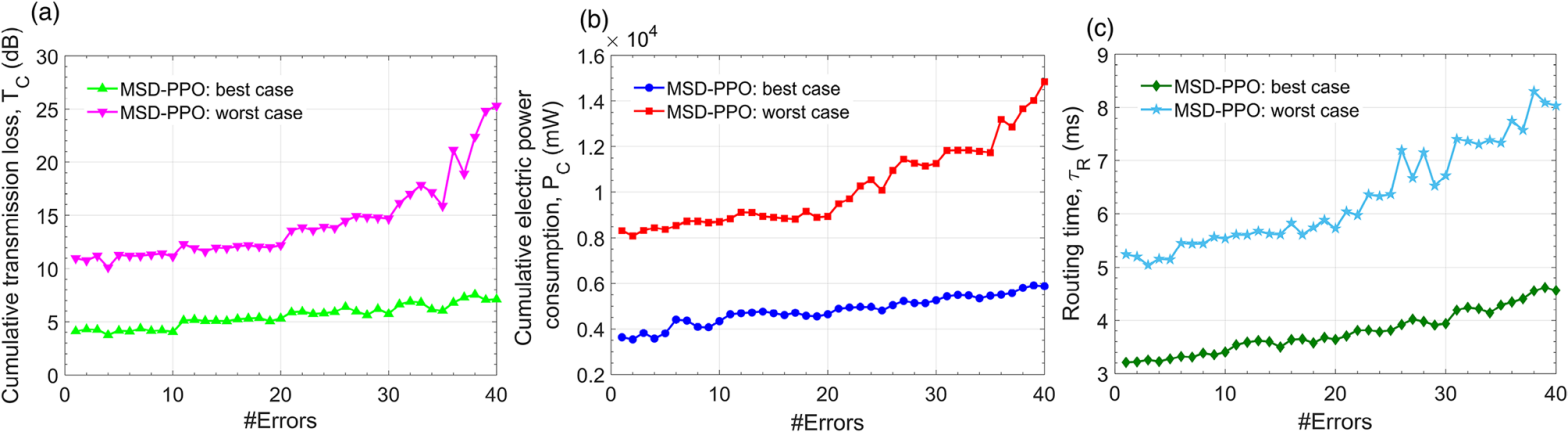
Performance results emulated by MSD according to the number of erroneous nodes in the physical environment for the best case and the worst case: **(a)** cumulative transmission loss, **(b)** cumulative electric power consumption, and **(c)** routing time.

**Figure 14:**
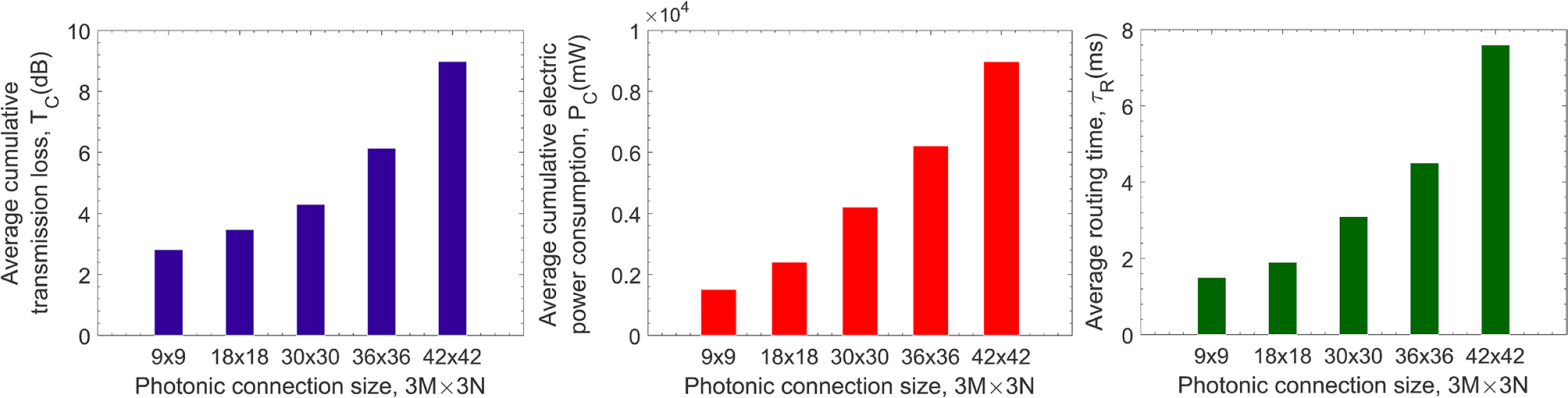
Performance of the learned routing policy as the size of the photonic chip network increases, in terms of transmission loss **(a)**, power consumption **(b)**, and routing time **(c)**.

#### Robustness to erroneous nodes

It is not unacceptable that MSD is more outperformed than that of reinforcement learning algorithms for optimization issues. To evaluate the effectiveness of different routing strategies and the proposed reinforcement learning algorithm, we need to consider the performance of the MSD deep learning model to events and responses from the physical environment of the on-chip integrated photonic network from the worst to the best cases after a successful training process. This issue is vital because a CMOS process for manufacturing a monolithic silicon-photonic chip network can be imperfect, or some fundamental chip units can be malfunctions over the timeline. The photonic chip network size is installed as M and N are all equal 36 exhibiting such commodious space that brute-force or heuristic algorithms become invalid or insufficient. Subfigures in Fig. 13 present the cumulative transmission loss $T_C$ (Fig. 13a), the electric power consumption $P_C$ (Fig. 13b), and the routing time $\tau _R$ (Fig. 13c) in the routing progress from a random pair from some input to some output versus the physical connection error for the MSD reinforcement learning model in the best and worst cases, respectively. The number of errors investigated randomly varies from 1 to 40. For the best case, the routing processes are efficient and linear increase versus three investigated performance factors thanks to the effective operation of advisors and the excellent environment discovery ability of the Sample Extraction Buffer element. For the worst case, when the error number is smaller than 20, the model still performs the routine effectively. The situation becomes different when the error number is greater than 20 that there is a clear distinction when MSD has difficulty in routing optical paths because all performance parameters become more frequently fluctuated. For the cumulative transmission loss, one can see that the transmission loss may take 4 dB to 7 dB if fortunately to meet the shortest path, for example, adjacent input-output pairs. However, even if it, unfortunately, meets the worst case, MSD still exhibits an ability to route effective optical paths thanks to the stable convergence and the practical feasibility of the off-policy. Because, as can be seen in Fig. 13, in the worst case with the longest path, the cumulative transmission loss is below 25 dB, and this value is within the allowable sensitive range for the current semiconductor photodetectors. Besides, the cumulative electric power consumption is about 3.6 W for the best case and not exceeded 15 W for worst-case. Furthermore, the routing time is below 8.4 ms in the worst-case. This is, therefore,appropriate for real-time routing in photonic connection networks. This effectiveness to erroneous numbers demonstrates that our chip network design has high stability and attains a large erroneous tolerance.

#### Scalability

To understand the scalability of the proposed PCN and the routing algorithm, we increase the size of the PCN from $8{\times }8$ to $9{\times }9$, $18{\times }18$, $36{\times }36$, and $42{\times }42$, as illustrated in Fig. 14. When size the PCN is $9{\times }9$, the resulting transmission loss, power consumption, and routing time are small, being 2.8 dB, 1512 mW, and 1.4 ms, respectively. With a considerably larger expansion up to $42{\times }42$, an excellent result was recorded with an approximation of 8.8 dB of transmission loss, 8763 mW of power consumption, and 7.6 ms of routing time. This scalability demonstrates that our chip network design is highly modular, and the routing policy is highly scalable, which is suitable for creating sizeable inter-chip communication networks and photonic data centers in the future.

## Broader impact

This article proposed a rectangular full-grid silicon photonic-on-chip network architecture enabling the four-degree connection/switching ability through the presence of the novel waveguide cross-connect structure. The original idea of this structure can be analogously manipulated to construct higher-degree photonic switches and more complicated network topology architecture as well as scale up a vast of connection i/o ports. Besides, the networks can be self-controlling after being completely trained by our hybrid deep reinforcement learning models can make the proposed PCN more effective in the routing optimization and multiple controlling workers of the optical paths to achieve high performances against the dynamic changes of traffic, connection quantity, and erroneous nodes in the network. Thus, this paper is more beneficial to a wide variety of PCNs based applications in terms of transparency, adaptivity, responsibility, and optimality, including reconfigurable optical add drop multiplexers in optical transmission nodes, all photonic routers in Petascale data centers, fully connected layers in photonic convolutional neural networks.

The proposed algorithm presenting in this investigation can be applied to control and optimize network resources and properties in many different topologies of distributed optical fiber communication networks, such as dynamic connection/node quantity, automatic traffic protection switching, optimal routing, ultrafast channel connection permutation restoration, sufficient energy consumption, and automatically updated configuration^91,92^. In such perspective scenarios, self-learning capability hidden in the control and administrative planes at distributed communication networks can execute and resolve better adaptive network configuration and resource management tasks via the deductive sampling capability associated with the Sample Extraction Buffer for the MSD-PPO algorithm of deep reinforcement learning model.

## Conclusion

We have proposed a full-grid photonic switching network based on novel multi-degree silicon photonic switches enabling the routing strategy operation via artificial intelligence techniques. The network architecture provides flexible bandwidth configuration for high performance while being energy efficient. In addition to having low physical cost and high energy efficiency, optimizing the transmission loss and power consumption in a massive range stands out as a key challenge. The routing strategy, which can be seamlessly incorporated into the switch controller, potentially provides an additional advantage for the physical layer performance optimization at no extra cost. An enhanced technique of the PPO algorithm thanks to applying multi-sample discovery agents into PPO exhibits significant results for routing strategy. By defining the number of global input-output in the switch in topologies, we reveal their optimal paths based on the current state of the photonic-on-chip network. Our analysis shows that the optical routing effectiveness of transmission loss, power consumption, and routing time when applying MSD based reinforcement learning. This routing strategy also demonstrates an excellent efficiency for erroneous network nodes and fabrication tolerance error, thus increasing the photonic network’s operating stability. Furthermore, our results show the scability of the network capacity demonstrated via both simulation and test platforms, even for moderate-scale silicon switches. Such marvelous properties make the proposed silicon photonic-on-chip networks being self-controlling in use and providing a potential for applications in decentralized petabyte data centers, photonic neural networks, big data processing, high-performance computing, and ultrafast optical intrachip communication (Suppl. Information).

## Supplementary Information

Supplementary Figures.
