# Supplementary material for: Self-controlling photonic-on-chip networks with deep reinforcement learning
Source: Sci Rep. 2021 Nov 30;11:23151. doi: 10.1038/s41598-021-02583-7 (PMC8632908; doi:10.1038/s41598-021-02583-7)
Supplement: Supplementary file 1 — Supplementary Figures. [file 41598_2021_2583_MOESM1_ESM.pdf]

Supplementary

This supplemetaty provides the link to Video that illustrates how our proposed architecture & rounting algorithms works at different scales as well as the link to data and source code of the MSD-PPO. In addition, this would provide the step-by-step instructions of how to run the code.

Link to video on Youtube: https://www.youtube.com/watch?v=o3Rl_cx3nnc

Link to source code (MSD-PPO) and Data: https://github.com/nguyendohoangkhoi/MSDPPOPCN

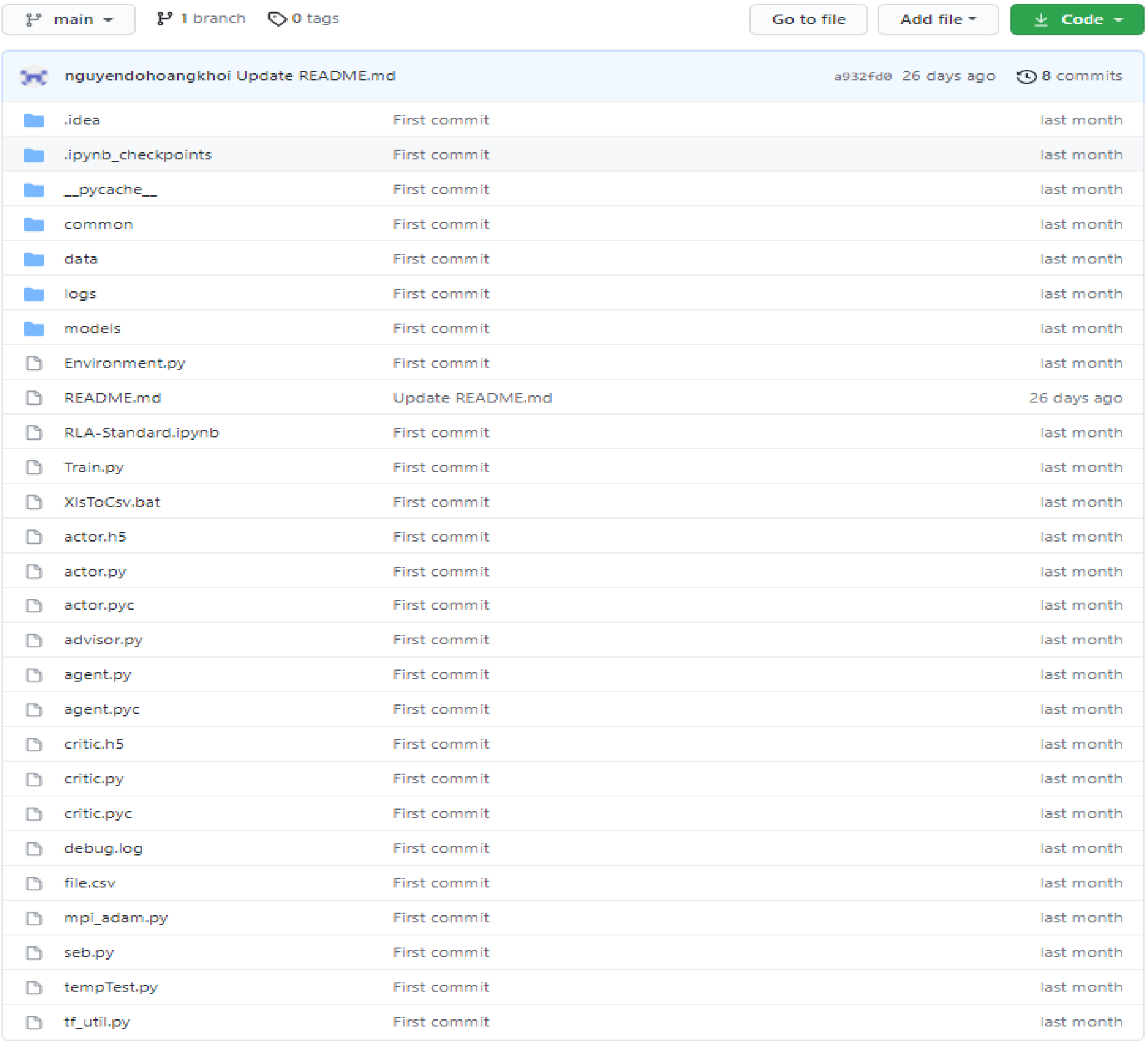

Supplementary figure S1. Source code and data folder

Multi-Sample Discovery Proximal Policy Optimization

Our data is included in "data" folder. A switch has four directions corresponding to the I, J, O, K. Each direction has three switches, for example, I1, I2, I3. Therefore, the format name of the data is set "NamePort_to_NamePort". Full detail implementation is in RLA-Standard.ipynb file.

Lamda	Output	Loss1	Loss2	pc
1.525	8.18E-02	3.12E-05	1.53E-04	192.5
1.53	1.49E-01	3.44E-05	2.43E-04	192.5
1.535	2.74E-01	4.58E-05	1.26E-03	192.5
1.54	4.81E-01	3.67E-05	2.28E-03	192.5
1.545	6.45E-01	9.57E-06	2.37E-03	192.5
1.55	6.52E-01	4.23E-06	2.53E-03	192.5
1.555	5.02E-01	1.28E-06	2.78E-03	192.5
1.56	2.93E-01	3.14E-07	1.90E-03	192.5
1.565	1.36E-01	1.97E-06	4.78E-04	192.5

Supplementary figure S2. Data format

In our work, we investigate the PCN with the wavelength in range from 1.525 to 1.565 (first collum). The output (transmission), loss_i (crosstalk) collum is to compute the reward which is the tranmission loss for the agent. The pc collum is to computer power consumption for each switch. The tranmission loss is computed using the equation (17) in the paper.

Code Usage

The model of Actor, Advisor, Critic and SEB are implemented in actor.py, advisor.py, critic.py and seb.py

For more detail about our implementation, please go to notebook file (RLA-Standard.ipynb) to follow step by step. We describe how to implement the map of PCN including changing map size, define reward, how the map work and the MSD-PPO algorithm in PCN etc.

If you want to check the training process, you can use tensor board by the command: "tensorboard --logdir=logs/"

In the case you want to use MSD-PPO with your customized dataset. You can put all your dataset in "data" folder with our format. Notice that if the data is xlsx file, you should click "XlsToCsv.bat" to convert all your data to csv file.

In the case you want to custom the size of PCN. Use the code and put your size in get_map() function.

```
pcn = get_map()
pcn = pcn.astype(float)
pcn.shape
```

Supplementary figure S3. Modifying-map size function

To load the data, you can use this code in RLA-Stardard.ipynb. Note that you need to put all data files in the “data” folder and structure them into the mentioned format.

```
In [24]: df = {}
         pc={}
         for file in source_data :
           data = pd.read_excel(file,header=0)
           print(data)
           row = data.loc[data['Lamda']==1.5500].values[0][1:]
           file_name = file.split('.')[0]
           output_loss = row[0]
           power_cons = row[3]
           #print(output_loss)
           df[file_name] = np.log10(output_loss*4/3)
           pc[file_name] = power_cons
         df
```

```
   Lamda    Output         Loss1     Loss2     pc
0  1.525  0.081787  3.120494e-05  0.000153  192.5
1  1.530  0.148833  3.441380e-05  0.000243  192.5
2  1.535  0.274497  4.577745e-05  0.001259  192.5
3  1.540  0.481452  3.673484e-05  0.002275  192.5
4  1.545  0.644609  9.567780e-06  0.002368  192.5
5  1.550  0.651973  4.227260e-06  0.002534  192.5
6  1.555  0.501727  1.284361e-06  0.002775  192.5
7  1.560  0.292534  3.142490e-07  0.001900  192.5
8  1.565  0.136158  1.971773e-06  0.000478  192.5
   Lamda    Output     Loss1     Loss2      pc
0  1.525  0.059389  0.000091  0.000750  184.15
1  1.530  0.118085  0.000042  0.001807  184.15
2  1.535  0.227252  0.000081  0.001311  184.15
3  1.540  0.436841  0.000184  0.000703  184.15
4  1.545  0.618331  0.000121  0.000174  184.15
5  1.550  0.637778  0.000104  0.000162  184.15
6  1.555  0.493877  0.000050  0.000634  184.15
7  1.560  0.291929  0.000028  0.000859  184.15
8  1.565  0.126916  0.000021  0.000950  184.15
   Lamda    Output     Loss1         Loss2     pc
0  1.525  0.079659  0.000390  1.350025e-04  174.4
1  1.530  0.144708  0.000323  1.044313e-04  174.4
2  1.535  0.281011  0.001153  3.742872e-05  174.4
3  1.540  0.484184  0.001795  2.249174e-05  174.4
4  1.545  0.645909  0.002086  6.102188e-06  174.4
5  1.550  0.651711  0.002257  4.898336e-06  174.4
```

Supplementary figure S4. Loading and pre-processing data

The following code to send the information of the agent's taking an action to the environment:

```
In [34]: qpcn.act(LEFT1)
         #qpcn.act(RIGHT2)
         qpcn.act(DOWN3)  # move down
         qpcn.act(DOWN3)  # move right
         qpcn.act(RIGHT2)  # move right
         qpcn.act(DOWN3)  # move right
         qpcn.act(UP1)  # move up
         qpcn.act(DOWN3)  # move up
         qpcn.act(DOWN1)  # move up
         qpcn.act(DOWN1)  # move up
```

Supplementary figure S5. Photonic on Chip Network (PCN) takes an action received from the agent and changes the state.

To train the model, we can run the cell 39th in the .ipynb file.

In [39]:

```
TRAIN_ITEagentIONS = 50
MAX_EPISODE_LENGTH = 128
TRAJECtoRY_BUFFER_SIZE = 32
BATCH_SIZE = 16
RENDER_EVERY = 100
AGGREGATE_STATS_EVERY = 1

if __name__ == "__main__":
    os.chdir("D:/Study/RL/RLAChips-V11")
    img_height, img_width = pcn.shape
    env = Qpcn(pcn)
    EPISODES = 200000
    k_permutation = 100
    num_states = pcn.size
    state_dim = env.state_dim
    input_dim, output_dim = state_dim, num_actions
    lr, gamma, loss_clipping, c1, lamda = 1e-6, 0.92 , 0.2, 0.001, 0.95
    agent = Agent(input_dim, output_dim, lr, gamma, loss_clipping, c1,lamda,k_permutation)
    tensorboard = ModifiedTensorBoard(log_dir="logs/{}-{}".format(MODEL_NAME, int(time.time())))
    MODEL_NAME = "model"
    AGGREGATE_STATS_EVERY = 1
    ep_rewards=[]
    ep_loss = []
    ep_pw = []
    win_count = 0
    num_non_cell = 30
    supervision_factor = 0.3
    for e in range(1,EPISODES+1):
        agent_cell = random.choice(env.free_cells)
        non_available_cell = random.choices(env.free_cells,k=num_non_cell)
        target_cell = random.choice(env.free_target_cells)
        #target_cell = random.choice(env.free_cells)
        #target_cell = (34,33)
        state = env.reset(agent_cell,target_cell,non_available_cell)
#         for i in non_available_cell:
#             print(i,env._pcn[i[0]][i[1]+1])
#             time.sleep(2)
        r_sum = 0
        loss_sum =0
        reward_sum = 0
        power_sum = 0
        done = False
        if e % 200==0:
            clear_output(wait=True)
        for cnt_step in range(MAX_EPISODE_LENGTH):
#             show(env)
#             time.sleep(0.5)
            clear_output(wait=True)
            #get action from agent given state
            #print(state)
            state = np.reshape(state,(-1,state_dim))
            action,pi_vec =  agent.act(state)
            ran_supervised = np.random.uniform(0,1)
            if ran_supervised < supervision_factor:
                env.supervised = 1
                action = env.valid_actions()[0]
                env.supervised = 0
            #get s_,r,done
            env.current_action = action
            next_state, reward, done= env.act(action)
            next_state = np.reshape(next_state,(-1,state_dim))
            [illegible]
```

Supplementary figure S6. The code block for preparing and training model components.
